# Supplementary material for: Elevating Air Temperature May Enhance Future Epidemic Risk of the Plant Pathogen Phytophthora infestans
Source: J Fungi (Basel). 2022 Jul 30;8(8):808. doi: 10.3390/jof8080808 (PMC9410326; doi:10.3390/jof8080808)
Supplement: Supplementary file 1 [file jof-08-00808-s001.zip › jof-1806638-supplementary.pdf]

Table S1 Geographic coordinate, annual mean temperature (AMT) and annual accumulative rainfall (AAR) in the seven locations sampled for *Phytophthora infestans*.

Population	Altitude(m)	Longitude	Latitude	Area(km^2)	AMT (°C)	AAR (mm)
Ningxia	1778	106°14'	36°01'	10523	7.00	300
Gansu	2089	105°43'	34°35'	14277	11.70	500
Guizhou	1330	105°56'	26°16'	9267	14.70	851
Yunnan	2677	102°43'	25°03'	21013	15.60	1100
Xiapu	31	119°59'	26°54'	13433	20.30	1450
Fuzhou	10	119°17'	26°05'	12255	20.50	1400
Guangxi	78	108°22'	22°50'	22245	22.60	1750

Table S2 Analysis of variance (ANOVA) for adaptation to aggressiveness among the *Phytophthora infestans* populations sampled from seven locations in China.

Source	DF	Sum of squares	Mean square	*F* value	*P*
Population	6	2826.07	471.01	22.15	<0.0001
Isolates	133	28855.43	216.96	10.2	<0.0001
Temperature	4	97333.27	24333.32	1144.06	<0.0001
Isolates*temperature	532	66367.79	124.75	5.87	<0.0001
Population*temperature	24	8879.72	369.99	17.4	<0.0001
